# Optimally splitting cases for training and testing high dimensional classifiers

**DOI:** 10.1186/1755-8794-4-31

**Published:** 2011-04-08

**Authors:** Kevin K Dobbin, Richard M Simon

**Affiliations:** 1 Department of Epidemiology and Biostatistics, College of Public Health, University of Georgia, Athens, GA, USA; 2 Biometric Research Branch, National Cancer Institute, National Institutes of Health, Rockville, MD, USA

## Abstract

**Background:**

We consider the problem of designing a study to develop a predictive classifier from high dimensional data. A common study design is to split the sample into a training set and an independent test set, where the former is used to develop the classifier and the latter to evaluate its performance. In this paper we address the question of what proportion of the samples should be devoted to the training set. How does this proportion impact the mean squared error (MSE) of the prediction accuracy estimate?

**Results:**

We develop a non-parametric algorithm for determining an optimal splitting proportion that can be applied with a specific dataset and classifier algorithm. We also perform a broad simulation study for the purpose of better understanding the factors that determine the best split proportions and to evaluate commonly used splitting strategies (1/2 training or 2/3 training) under a wide variety of conditions. These methods are based on a decomposition of the MSE into three intuitive component parts.

**Conclusions:**

By applying these approaches to a number of synthetic and real microarray datasets we show that for linear classifiers the optimal proportion depends on the overall number of samples available and the degree of differential expression between the classes. The optimal proportion was found to depend on the full dataset size (n) and classification accuracy - with higher accuracy and smaller *n *resulting in more assigned to the training set. The commonly used strategy of allocating 2/3rd of cases for training was close to optimal for reasonable sized datasets (*n *≥ 100) with strong signals (i.e. 85% or greater full dataset accuracy). In general, we recommend use of our nonparametric resampling approach for determing the optimal split. This approach can be applied to any dataset, using any predictor development method, to determine the best split.

## Background

The split sample approach is a widely used study design in high dimensional settings. This design divides the collection into a training set and a test set as a means of estimating classification accuracy. A classifier is developed on the training set and applied to each sample in the test set. In practice, statistical prediction models have often been developed without separating the data used for model development from the data used for estimation of prediction accuracy [1]. When the number of candidate predictors (*p*) is larger than the number of cases as in microarray data, such separation is essential to avoid large bias in estimation of prediction accuracy [2]. This paper addresses the question of how to optimally split a sample into a training set and a test set for a high dimensional gene expression study, that is, how many samples to allocate to each group.

Two approaches to evaluating splits of the data are examined. The first approach is based on simulations designed to understand qualitatively the relationships among dataset characteristics and optimal split proportions. We use these results also to evaluate commonly used rules-of-thumb for allocation of the data to training and test sets. Our second approach involves development of a non-parametric method that does not rely on distributional assumptions and can be applied directly to any existing dataset without stipulating any parameter values. The nonparametric method can be used with any predictor development method (e.g., nearest neighbor, support vector machine).

This paper addresses the situation in which the accuracy of a predictor will be assessed by its performance on a separate test set. An alternative approach is to apply resampling-based methods to the whole dataset. Because re-sampling strategies have been commonly mis-used, often resulting in highly biased estimates of prediction accuracy [2,3], many journals and reviewers mis-trust cross-validation and require validation on a sample not used for model development. Another advantage of the split sample method, particularly in large collaborative studies in which multiple groups will be developing predictors, is that the test set can be kept under "lock and key" by a honest broker [4].

The question addressed in this paper has not to our knowledge been addressed before. Sample splitting has been addressed in other contexts, such as comparing different *k*-fold cross validations [5] or developing hold out estimation theory [6] and bounds on Bayes error [7]. Mukherjee et al. [8], Fu et al. [9], and Dobbin and Simon [10] developed methods for planning the size of a training set, but these methods do not address the allocation of cases in an existing dataset to training and test portions. Since many gene expression based classifiers are developed retrospectively, there is often little control of the sample size.

In the next section we describe the parametric modeling approach and the nonparametric approach that can be applied to specific datasets. We also present the results of application of these methods to synthetic and real world datasets. In the Conclusions section, recommendations for dividing a sample into a training set and test set are discussed.

### Approach

The classifier taken forward from a split-sample study is often the one developed on the full dataset. This full-dataset classifier comes from combining the training and test sets together. The full-dataset classifier has an unknown accuracy which is estimated by applying the classifier derived on the training set to the test set. The optimal split will then be the one that minimizes the mean squared error (MSE) with respect to this full-dataset classifier. The MSE naturally penalizes for bias (from using a training set smaller than *n*) and variance.

#### MSE decomposition

In the supplemental material [Additional file 1: Supplemental Section 1.2], it is shown that under mild assumptions the MSE is proportional to(1)

**Figure 1:**
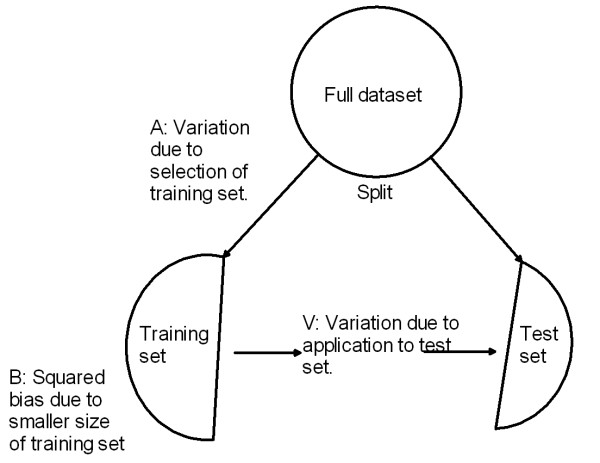
**Conceptual Diagram**. Diagram of mean squared error decomposition.

Here we have symbols *A*, *V *and *B *to depict the decomposition, and these are used throughout the discussion below. Here is a description of each term in Equation (1). Figure 1 shows the breakdown visually.

##### A = Accuracy Variance Term

The first term in Equation (1) reflects the variance in the true accuracy of a classifier developed on a training set  selected from the full dataset . Not all training sets  will result in predictors with exactly the same accuracy. The variation in actual (true) accuracy among all these different predictors is the *A *term.

##### V = Binomial Variance Term

The second term in Equation (1) is the variance in the estimated accuracy that results from applying the classifier to the test set. This is a binomial variance because the classifier developed for a specific training set has some fixed true accuracy (success probability), and there are *n - t *independent samples represented in the test set.

##### B = Squared Bias Term

The third term in Equation (1) is the squared bias that results from using a classifier that was developed on *t *training samples to estimate the accuracy of a classifier which is developed on *n *samples.

#### Model-based simulations for high dimensional expression profiles

With each sample is associated a *p*-dimensional vector of log gene expression measurements, say *x*, which is assumed to follow the multivariate normal distribution with mean vector *μ*_1 _for class 1 and *μ*_2 _for class 2 and common covariance matrix Σ. Of the p genes, m are assumed differentially expressed with difference in mean expression levels between classes of 2*δ *and the remaining p-m genes are not differentially expressed. Extensive simulations under a variety of conditions indicated that the components of MSE depended on the separation of the classes with regard to gene expression and this is determined by the number of differentially expressed genes, the degree of differential expression and the correlation among the differentially expressed genes. In general none of these quantities are known before analyzing the data. However, we have attempted to utilize extensive simulation results to understand the relationship between sample size, class separation and splitting effectiveness in order to provide robust general recommendations.

Our simulations use the compound covariate predictor [11], with gene selection performed using the optimal selection cutpoint algorithm described in Dobbin and Simon [10]. We adjusted the method in Dobbin and Simon [10] for predicting an optimal significance level for gene selection to avoid assuming that the prevalence of the classes is known.

The MSE as a function of splitting proportion is estimated for each simulated dataset in the following way:

1. Given 2*δ/σ *standardized fold change, *m *informative genes, dimension *p*, *n *samples available, and a covariance matrix Σ, generate a dataset *S* from the probability model. Randomly select R training sets of size *t*. A grid of t values are evaluated for each total sample size n.

2. For each *t *above, calculate the optimal significance level cutoff *α *to use for gene selection [10].

3. Using the optimal *α *levels to select genes from pooled variance t-tests, develop compound covariate predictors (CCP) [11] for each training set.

4. For each classifier developed on a training set of size t, apply the classifier to the corresponding test set of size n-t and estimate the classification accuracy. Average estimates over the R replicates to obtain the mean predicted accuracy estimate.

5. Develop a CCP classifier on the full dataset *S *of n cases. Using the parameters used to generate *S*, the true accuracy of the classifier developed on the full dataset was computed from theory.

#### Simulation approach with empirical effect sizes and covariance matrix from real microarray dataset

In order to simulate from a model reflecting more closely real microarray data, data were generated from class *C*_1 _and *C*_2 _as *Normal *where  and  were estimated from the dataset of Rosenwald et al. [12]. Specifically,  where *S *is the sample covariance matrix (pooled over the classes), and *Diag*(*S*) is a matrix of zeros except for the diagonal, which is equal to the diagonal of *S*. The covariance matrix was shrunk away from singularity using *p *= 0.90 and *p *= 0.60. Then, elements of  were estimated empirically as described in the table legend. Finally, datasets were generated from the model.

Full dataset accuracies were computed using the equation  where *k *is the classification cutpoint [13]. Datasets were split to obtain the test set/training set accuracy estimate. Empirical MSE's were calculated.

#### A method for determining the optimal sample split for a particular dataset, which utilizes a nonparametric data re-sampling approach

The nonparametric bootstrap method of estimating standard error [14] was used to estimate the variance of the performance of a predictor developed on a training set of size *t *and applied to a test set of size *n - t*. In our previous notation, this was *A *+ *V*. Splitting was performed prior to resampling in order to avoid overlap between the training and test sets.

In order to estimate the squared bias term *B *we considered adopting learning curve methods [15], as used previously in Mukherjee et al. [8]. Briefly, [8] uses a parametric nonlinear least squares regression approach that fits a learning curve model to datapoints of the plot with training set size *t *on the x-axis and the estimated error rate on the y-axis. Fitting a learning curve of the form *e *= *a *+ *b/t^α ^*where *e *is the expected error and *t *is the training set size (and *α *> 0), provides an estimate of the asymptotic error rate (*a*), i.e., when *t *= ∞. However, we found the parametric learning curve model for the data often did not fit our simulated or real data adequately. Also, estimation of the squared bias term *B *does not require estimation of the asymptotic error (*a*), but only the mean error rate for limited training sizes *t *≤ *n*. So instead, we use a nonparametric smoothing spline to fit the plot with the training sample size *t *on the x-axis and the average error rate on the y-axis. When the learning curve raw data were not monotone (usually because the error rate had stopped decreasing significantly relative to the noise level present), then we used isotonic regression to force monotonicity of the fitted curve.

The squared bias term is estimated as follows:

1. For fixed *n*, and for *t *= 10, 20,..., *n - *10, randomly divide the dataset into a training set and a test set 1,000 times.

2. For each *t*, develop a classifier on each of the 1,000 training sets and apply the classifier to the corresponding test set. For each *t*, calculate the mean error rate *w *of these 1,000 classifiers.

3. Fit a smoothing spline or isotonic regression of *w *on *t *using spline case weights 1/*t*. Adjust degrees of freedom visually based on the smoothing spline plot.

4. For *t *= 10, 20,..., *n*, calculate, the fit-value from the spline or isotonic regression of the error rate on *t*.

5. Estimate the squared bias using .

## Results and Discussion

We applied the parametric method to high dimensional multivariate normal datasets, while varying the parameter settings and the class prevalences. Results are shown in Table 1 and [Additional file 1: Supplemental Table S1]. We considered total samples of size *n *= 200, *n *= 100 and *n *= 50. For example, when *m *= 50 genes are informative and *n *= 200, then the optimal number of samples for the training set (reading across the first row of Table 1) is 170, 70 or more, 30 or more, and 20 or more for effect sizes of 0.5, 1.0, 1.5 and 2.0, respectively. The "or more" in the last three training set sizes indicates that training set sizes anywhere from the specified size up to 190 result in practically equivalent mean squared error.

**Table 1:** Table of optimal allocations of the samples to the training sets

Optimal number to training set				
**n **= **200**				

	**Effect = 0.5**	**Effect = 1**.**0**	**Effect = 1.5**	**Effect = 2.0**

**DEG = 50**	170 (86%)	70+ (>99%)	30+ (>99%)	20+ (>99%)

**DEG = 10**	150 (64%)	130 (94%)	100 (99%)	60+ (>99%)

**DEG = 1**	10 (52%)	150 (69%)	120 (77%)	80 (84%)

**n **= **100**				

**DEG = 50**	70 (64%)	80 (>99%)	30+ (>99%)	20+ (>99%)

**DEG = 10**	10 (55%)	80 (91%)	70 (99%)	40+ (>99%)

**DEG = 1**	10 (51%)	40 (63%)	80 (77%)	70 (84%)

**n **= **50**				

**DEG = 50**	10 (59%)	40 (99%)	30+ (>99%)	20+ (>99%)

**DEG = 10**	10 (52%)	40 (78%)	40 (98%)	40 (>99%)

**DEG = 1**	10 (50%)	10 (54%)	30 (71%)	40 (83%)

Entries in table are  where *t *is the optimal number for the training set and *Acc *is the average accuracy for a training set of size *n*. Total sample size is *n*. "DEG" is the number of independent differentially expressed genes. "Effect" is the standardized fold change for informative genes (difference in mean expression divided by standard deviation). Notation such as "50+" indicates that the MSE was flat, achieving a minimum at t = 50 and remaining at that minimum for *t *> 50. (Here, "flat" is defined as having a range of MSE values less than 0.0001.) Data generated with dimension *P *= 22,000. Each table entry based on 1,000 Monte Carlo simulations. Equal prevalence from each of two classes.

Several features are apparent in Table 1: (i) when the achievable accuracy is not much greater than 50%, the optimal split allocates the vast majority of samples to the test set. In this circumstance, no good classifier is possible so additional samples allocated to the training set are wasted and detract from lowering the variance of estimation in the test set; (ii) when the gene expression profiles of the two classes are widely separated, e.g., with a large number of differentially expressed genes and large effect sizes, small training sets are adequate to develop highly effective classifiers. The MSE is flat in this circumstance nor large test sets are needed.

[Additional file 1: Supplemental Table S1] shows the results when the prevalence is unbalanced, namely, 2/3 from one class and 1/3 from the other class. The results for this imbalanced prevalence setting are very similar to the equal prevalence setting. This suggests that the same general optimal splits apply for a range of class prevalence (33% to 67%).

**Figure 2:**
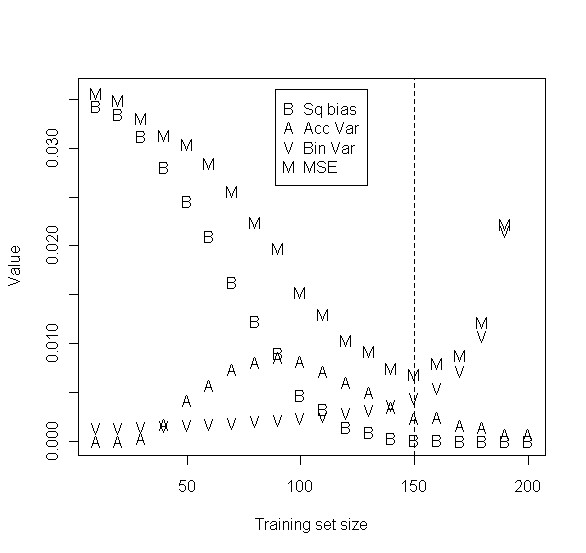
**Example of MSE decomposition**. Example figure showing the relative contributions of the three sources of variation to the mean squared error. This is a scenario from one entry in Table 1. Plots for all other scenarios associated with Table 1 and [Additional file 1: Supplemental Table S1]. Here there is m = 1 informative gene, n = 200 total samples available for study, and the standardized fold change for the informative gene is 2*δ*/*σ *= 1.0.

The relative sizes of the three terms contributing to the mean squared error of Equation (1) for the scenarios of Table 1 and [Additional file 1: Supplemental Table S1] are shown in the Supplementary material [Additional file 1]. An example is shown in Figure 2. Generally, the A term tends to be relatively small across the range of sample sizes.

The squared bias term B tends to be relatively large for small sample sizes and to dominate the other terms. When development of a good classifier is possible, the actual accuracy of classifiers developed on the training set may initially increase rapidly as the training set size increases. As the sample size increases, the bias term B decreases until no longer dominating. This is because the accuracy of the classifier improves as the size of the training set increases and approaches the maximum accuracy possible for the problem at hand. The rate of decrease of the squared bias term B will depend somewhat on the type of classifier employed and on the separation of the classes. When the classes are not different with regard to gene expression, learning is not possible and B will equal zero for all training set sizes.

The binomial variance term V is generally relatively small unless the test set becomes very small at which point it often dominates. The exceptions to this general rule are in cases where the prediction accuracy nears 1 for *t *<*n*, in which case this V term remains near zero even as the test set size becomes small. Another partial exception is when the full dataset accuracy is below 85%, when the binomial variance increases.

**Figure 3:**
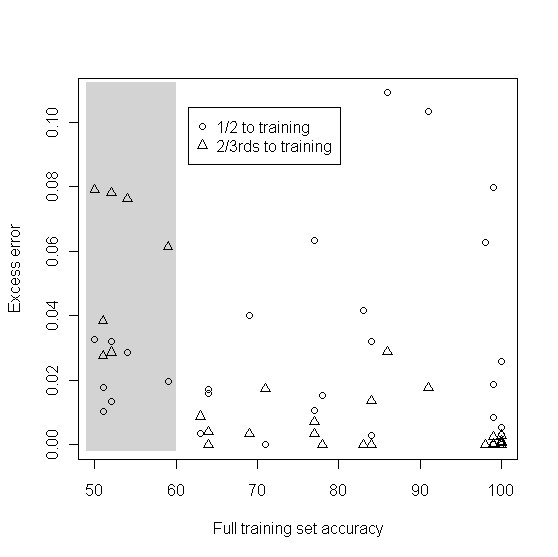
**Comparing two rules of thumb**. Comparison of two common rules-of-thumb: 1/2 the samples to the training set and 2/3 rds of the samples to the training set. X-axis is the average accuracy (%) for training sets of size n. "Excess error" on the y-axis is the difference between the root mean squared error (RMSE) and the optimal RMSE. Each point corresponds to a cell in Table 1. Gray shading indicates scenarios where mean accuracy for full dataset size is below 60%.

Figure 3 is a comparison of the two most common rules of thumb for splitting a sample into a training set and a test set. The figure compares 50% allotment to the training set versus 67% allotment to the training set for the equal prevalence case. Each scenario represented in Table 1 is also present in Figure 3. The x-axis is the average accuracy (%) for classifiers developed from the full dataset of *n *samples. The y-axis is the excess error from using a non-optimal split. The discussion is organized around the full dataset accuracy:

• When the achievable true accuracy using the full dataset for training is very close to 1, both the 50% allotment and the 67% allotment to the training set result in similar excess error.

• When the achievable true full dataset accuracy is moderate, say between 60% and 99%, then in several cases, assigning 67% to the training set results in noticeably lower excess error, while in other cases the two allotment schemes are roughly equivalent.

• Finally, and not surprisingly, when the achievable true full dataset accuracy is below 60% (shaded area on graph), then allotment of 50% to the training set is preferable.

In sum, this graph shows that allotment of 2/3 rds to the training set is somewhat more robust than allotment of 1/2 to the training set.

The nonparametric method was applied to simulated datasets and the MSE estimates compared to the parametric approach. Agreement between the two was very good [Additional file 1: Supplemental Section 4].

Table 2 and [Additional file 1: Supplemental Section 5] shows that the results are similar under an empirically estimated covariance matrix and distance between the classes [16-18].

**Table 2:** Empirically estimated effects and covariance

*p*	**Bayes Acc**.	*n*	**Prev**.	%*t*	Full data Accuracy	Opt. Vs. *t *= 2/3	Opt. Vs. *t *= 1/2
0.9	0.962	240	50%	58.3	0.961	0.001	0.002
0.6	0.861	240	50%	54.2	0.860	0.003	0.002

Simulation results based on empirical estimates of covariance matrix and effect sizes. Columns are: *p *is the weight on a diagonal matrix, Bayes Acc. is the optimal accuracy possible, *n *is the total sample size, Prev. is the prevalence from the most prevalent group, %*t *is the optimal allocation proportion to training, Full data Accuracy is the mean accuracy when *n *= 240, and Opt. vs t = 2/3 is the root mean squared difference (RMSD) for the optimal rule and the 2/3 rds-to-training rule, and Opt vs t = 1/2 is the RMSD between the optimal rule and the 1/2-to-training rule. Sample covariance matrix *S *calculated from [12]. Effect sizes are estimated by the Empirical Bayes method of [10] with effect sizes shrunk to 80% of the empirical size. We followed methods similar to those previously proposed ([16], [17], [18]) to obtain non-singular covariance matrix estimates, namely , where *diag*(*S*) is a matrix of zero's and diagonal elements of *S*. Bayes accuracy is the optimal accuracy for a linear classifier in the population, which is (e.g., [13] where  is a vector of half-distances between the class means. The number of informative genes was selected to achieve realistic Bayes (optimal) accuracies, so that all other gene effects were set to zero. Genes with largest standardized fold changes were selected as informative.

Table 3 shows the results of the application of the nonparametric method to several real-world datasets.

**Table 3:** Applications to real datasets

Dataset	*n*	Prevalence	%*t*	Full dataset accuracy	Optimal vs.	Optimal vs.
Rosenwald	240	52%	63%	0.96	0.001	0.002
Boer	152	53%	53%	0.98	0.004	2e-4
Golub	72	65%	56%	0.95	0.002	0.004
Sun	131	62%	31%	0.83	0.022	0.008
van't Veer	117	67%	26%	0.78	0.004	0.001

Nonparametric bootstrap with smooth spline (or isotonic regression) learning curve method results [Additional file 1]. *n *is the total number of samples from the two classes, and "Prevalence" is the prevalence of the majority class. %*t *is the percent of samples allocated to the training set under optimal allocation, *t/n *·100%. "Full dataset accuracy" is the estimated mean accuracy on the full dataset of size *n*. "Optimal vs.  rule" is the difference between the root mean squared error for an optimal training set allocation and for the "2/3 rds to training set" allocation rule. The rightmost column is for the "1/2 to training set" allocation rule. Classes for datasets are: Germinal Center B-cell-like lymphoma versus other (Rosenwald et al., 2002), renal clear cell carcinoma primary tumor versus control normal kidney tissue (Boer et al., 2001), acute myelogenous leukemia versus acute lymphoblastic leukemia (Golub et al., 1999), glioblastoma versus oligodendroglioma (Sun et al., 2006), grade 1/2 versus grade 3 lung cancer (van't Veer et al., 2002).

Note that the rightmost two columns show the excess error when 1/2 and when 2/3 rds are allotted to the training set. For the Rosenwald et al. [12] dataset of diffuse large B-cell lymphoma, we estimated the optimal split for distinguishing between germinal-center B-cell-like lymphoma from all other types. For this dataset of *n *= 240 patient samples, the optimal split was 150 : 90, with about two-thirds of the samples devoted to the training set. The excess error (root mean square error difference, RMSD) from the 2/3 rds to training set rule of thumb is 0.001; as a comparison, the RMSD for a simple binomial random variable (with p = 0.96) between a sample size of 236 and 240 is also 0.001. Hence, the excess error at *t *= 2*n*/3 is very small.

For the Boer et al. [19] dataset, the optimal split was 80 for the training set and 72 for the test set, so that 53% were used to train the classifier to distinguish normal kidney from renal cell carcinoma. The dramatic difference in gene expression between cancer and normal tissues meant that a smaller training set size was needed to develop a highly accurate classifier [Additional file 1: Supplemental Section 6.3]. As a result, the 1/2 to training set rule of thumb is a little better than the 2/3 rds to training split. That being said, the excess error when 2/3 rds ares used for training is only 0.004. For comparison, the RMSD of 0.004 is similar to the RMSD resulting from increasing the sample size from 142 to 152 in simple binomial sampling (when *p *= 0.98).

For the Golub et al. [20] dataset, the optimal split was 40 for the training set and 32 for the test set, or 56% for the training to distinguish acute lymphoblastic leukemia from acute myologenous leukemia. This is another example of two classes with dramatically different expression profiles. Like the Rosenwald dataset, the 2/3 rds to training set rule resulted in smaller excess error than the 1/2 rule.

To distinguish oligodendroglioma from glioblastoma in the the Sun et al. [21] dataset required 40 for the training set and 91 for the test set, or 31% for the training set. This optimal training sample size was somewhat smaller than expected. This appeared to be due to the accuracy leveling off after *t *= 40 training samples, while the variance terms increased monotonely for *t *> 40. The multidimensional scaling plot for these data [Additional file 1: Supplemental Section 6.4] showed a pronounced separation into two groups of cases - but these groups only partly corresponded to the class labels. The two groups were found easily with n = 40 samples, but the corresponding error rate was relatively high because of the imperfect correlation between the class labels and the two clusters in the plots. One is left to speculate whether this pattern was the result of real underlying biology, or artifacts such as batch effects or sample labeling errors. In this case it did appear that 40 samples in the training set was adequate to achieve accuracy near the best possible with the full n = 130 samples.

A possible explanation for the Sun et al. [21] dataset is that the full dataset accuracy was relatively low. We therefore investigated another dataset of van't Veer et al. [22] which also had low full dataset predictive accuracy and found a similar pattern. As shown [Additional file 1: Supplemental Section 6.5], the multidimensional scaling plot of grade 1/2 lung tumors versus grade 3 lung tumors showed two groups that did not match up with the tumor grade labels. This non-normality within groups may reflect underlying biological heterogeneity. As can be seen in the table, the optimal training set proportion is below 50% for this dataset as it was for the Sun et al. dataset, suggesting that with lower accuracies the setting is more complex and a single rule of thumb may not be adequate.

The supplement provides figures related to the fitting on the real datasets [Additional file 1: Supplemental Section 6]. We found that for the application to the real-world microarray datasets it was critical to perform at least 1,000 bootstrap re-samplings and 1,000 sample splits in order to obtain adequately de-noised MSE curves over the range of sample sizes.

## Conclusions

We have examined the optimal split of a set of samples into a training set and a test set in the context of developing a gene expression based classifier for a range of synthetic and real-world microarray datasets using a linear classifier. We discovered that the optimal proportion of cases for the training set tended to be in the range of 40% to 80% for the wide range of conditions studied. In some cases, the MSE function was flat over a wide range of training allocation proportions, indicating the near-optimal MSE performance was easy to obtain. In other cases, the MSE function was less flat, indicating clearer optimal selection. In general, smaller total sample sizes led to a larger proportions devoted to the training set being optimal. Intuitively this is because for a given degree of class separation, developing an effective classifier requires a minimal number of cases for training and that number is a greater proportion of a dataset with fewer total cases.

The number of cases needed for effective training depends on the "signal strength" or the extent of separation of the classes with regard to gene expression. "Easy" classification problems contain individual genes with large effects or multiple independent genes with moderately large effects. For such problems the potential classification accuracy is high (low Bayes error). The number of training cases required for near optimal classification for such datasets is smaller and hence smaller proportions devoted to the training set could be near optimal (for *n *= 100 - 200).

We found that when the average true accuracy of a classifier developed on the full dataset (size *n*) was >85%, then a  training-to-test set split resulted in near optimal MSE in all settings considered. Based on careful analysis and interpretation of the extensive simulations in the Appendix, we think that the rule of thumb that assigns 2/3*rds *to the training set and 1/3*rd *to the test set performs well in such situations. A separate Section in the Supplemental material describes the reasoning behind this recommendation. Generally, however, there will be uncertainty about the true full sample accuracy achievable and we recommend that the nonparametric resampling algorithm that we developed be applied to determine the optimal split. In applying this method the specific classifier of interest should be used. Use of our non-parametric algorithm to determine the optimal split, rather than one of the standard rules-of-thumb provides protection against the intra-class genomic heterogeneity that appears present in the Sun and van't Veer datasets.

Throughout the simulation studies, this paper has focused on common classifiers which are expected to perform well. Our simulation results should be applicable to the commonly used linear classifiers such as diagonal linear discriminant analysis, Fisher linear discriminant analysis and linear kernel support vector machines. However, there are many other types of classifiers that are currently being investigated. It is beyond the scope of this manuscript to comprehensively examine the MSE patterns of training set size variation for all these classifiers. The simulation results may not carry over to radically different types of classifiers, which may learn at a much different rate or have very different full dataset accuracies than those examined here. It is important not to over-interpret what is necessarily a limited simulation study.

This paper focused on the objective of obtaining a classifier with high accuracy. In some clinical contexts other objectives may be more appropriate, such as estimation of the positive and negative predictive values, or area under the ROC curve. If the prevalence is approximately equal for each class, however, then a high overall accuracy will be highly correlated with high negative and positive predictive values and AUC, so the guidelines here are likely to carry over to these other metrics.

The population prevalence from each class can be an important factor in classifier development. In this paper we looked at equal prevalence from each class, and at the case of 2/3 to 1/3 prevalence split in our simulations. The real datasets had prevalences within this range as well. In cases where there is significant prevalence imbalance between the classes (e.g., 90% versus 10%) there will often be a number of issues outside the scope of this paper. To modify our method for that context, one would need to address whether oversampling from the under-represented class is needed, and whether the cost of misallocation should differ by class.

We looked at a range of sample sizes from *n *= 50 to *n *= 200. In practice, sample sizes of *n *= 50 are probably too small to divide into a training set and a test set, and a better design uses resampling methods to estimate the classification accuracy instead. This study supports the general advice to use resampling methods in small sample settings because in these settings our method indicates that the MSE is generally minimized when most of the samples are devoted to the training set, with a typical allocation of 40 to training and only 10 samples for the test set. This will usually be inadequate except in very preliminary exploratory studies. For example, even if the observed classification accuracy in the test set is 10/10 = 100%, the 95% confidence interval for classification accuracy is 69% - 100%.

The data based resampling method presented in this paper can be used with any predictor development method by making minor modifications to the algorithm outlined in the Results.

## Methods

Computations were carried out in C++ using a Borland 5 compiler and Optivec 5.0 vector and matrix libraries, and R version 2.6.1 (including R "stats" package for smooth.spline and isoreg functions). Gene expression data were obtained from the BRB ArrayTools Data Archive for Human Cancer Gene expression (url: http://linus.nci.nih.gov/BRB-ArrayTools.html), except for [20] data which was retrieved from the Broad Institute website (url: http://www.broad.mit.edu/cgi-bin/cancer/datasets.cgi). Data were normalized using MAS 5.0 and median centering each array using either R or BRB ArrayTools (developed by Dr. Richard Simon).

## Authors' contributions

Both authors contributed to all aspects of manuscript development.

## Declaration of competing interests

The authors declare that they have no competing interests.

## Pre-publication history

The pre-publication history for this paper can be accessed here:

http://www.biomedcentral.com/1755-8794/4/31/prepub

## Supplementary Material

Additional file 1 **Article supplement**. Contains additional tables, figures, theoretical derivations and discussions. Click here for file
