# Supplementary material for: Optimally splitting cases for training and testing high dimensional classifiers
Source: BMC Med Genomics. 2011 Apr 8;4:31. doi: 10.1186/1755-8794-4-31 (PMC3090739; doi:10.1186/1755-8794-4-31)

Supplemental material for "Optimally splitting cases for training and testing high dimensional classifiers"

Kevin K. Dobbin and Richard M. Simon

1 Supplemental Section 1: Notation, proof, and further discussion

1.1 Notation

The total number of samples available is n. The number of samples in the training set is t and the number in the validation set is v with $n = t + v$. The training set is $\mathcal{T} = \{x_1, ..., x_t\}$, the validation set is $\mathcal{V} = \{x_{t+1}, ..., x_{n=t+v}\}$, and the entire dataset is $\mathcal{S} = \{x_1, ..., x_n\}$.

- $\widehat{A}(\mathcal{T}, \mathcal{S} - \mathcal{T})$ is the observed conditional accuracy of the split sample classifier. In other words, the proportion correctly classified by a classifier developed on the training set $\mathcal{T}$ when it is applied to the validation set $\mathcal{S} - \mathcal{T}$. This accuracy is a function of (conditional on) the samples chosen for S and how $\mathcal{S}$ is split into a trainin set, $\mathcal{T}$, and a validation set, $\mathcal{S} - \mathcal{T}$.
- $A(\mathcal{T})$ is the accuracy of the classifier developed on the samples $\mathcal{T}$ that would be observed if it were applied to the entire population of interest. $A(\mathcal{T})$ is a function of (conditional on) the samples chosen for T. McLachlan (1992) refers to this as the *actual accuracy* and Efron and Tibshirani (1997) as the *true accuracy*.
- Similarly, $A(\mathcal{S})$ is the conditional accuracy of the classifier developed on the full dataset.
- $\mu_A(t) = E\left[A(\mathcal{T})\right]$ is the expected (unconditional) accuracy of classifiers developed on training sets of size t, where the expectation is taken over training sets of size t in the population.
- Similarly $\mu_A(n) = E\left[A(\mathcal{S})\right]$ is the expected (unconditional) accuracy of classifiers developed on training sets of size n, where the expectation is taken over all sets of size n in the population.

1.2 Decomposition of the mean squared error

Let $\mathcal{S}$ represent a sample of size n taken from the population, and Ξ the set of all such samples in the population. Let $A(\mathcal{S})$ be the true (actual) accuracy of a classifier developed on a sample set $\mathcal{S}$; $A(\mathcal{S})$ is also called the "conditional accuracy" because it is conditional on the training set $\mathcal{S}$. Let $\hat{A}(\mathcal{T}, \mathcal{S}-\mathcal{T})$ be the split sample estimate of accuracy, estimated by splitting the sample into the training set $\mathcal{T} \subset \mathcal{S}$ and the validation set $\mathcal{S}-\mathcal{T}$. Then

$$MSE = E\left[\left(\hat{A}(\mathcal{T}, \mathcal{S}-\mathcal{T}) - A(\mathcal{S})\right)^2\right]$$

where the expectation is taken first over all $\mathcal{S} \in \Xi$ and then over all $\mathcal{T} \in \mathcal{S}$. The mean squared error is $E\left[\left\{\widehat{A}(\mathcal{T}, \mathcal{S}-\mathcal{T}) - A(\mathcal{S})\right\}^2\right]$, where the expectation is taken over all sets of size n in the population, and then conditionally over all divisions of each set of size n into a training set of size t and a validation set of size $n-t$.

$$\begin{aligned} E\left[\left(\hat{A}(\mathcal{T}, \mathcal{S}-\mathcal{T}) - A(\mathcal{S})\right)^2\right] &= E\left[\left(\hat{A}(\mathcal{T}, \mathcal{S}-\mathcal{T}) - A(\mathcal{T}) + A(\mathcal{T}) - A(\mathcal{S})\right)^2\right] \\ &= E\left[\left(\hat{A}(\mathcal{T}, \mathcal{S}-\mathcal{T}) - A(\mathcal{T})\right)^2\right] + E\left[(A(\mathcal{T}) - A(\mathcal{S}))^2\right] \end{aligned}$$

where we have assumed that the cross-term is zero.
Consider the first term. $A(\mathcal{T})$ is the true predictive accuracy of the classifier that was developed on the training set. Now technically $\mathcal{S}$ is fixed ahead of $\mathcal{T}$, so that $\mathcal{S}-\mathcal{T}$ is not a random sample from the population. But it is equivalent to a random sample since $\mathcal{S}$ is a random sample from the population and $\mathcal{T}$ a random sample from $\mathcal{S}$. Looked at this way, it can be seen that the first term is an average of accuracy variances from binomial samples of size $n-t$. We therefore call this first term the binomial variance term V and it is approximately equal to:

$$\begin{aligned} E\left[\left(\hat{A}(\mathcal{T}, \mathcal{S}-\mathcal{T}) - A(\mathcal{T})\right)^2\right] &= E\left[\frac{A(\mathcal{T})(1-A(\mathcal{T}))}{n-t}\right] \\ &\approx \frac{\mu_A(t)[1-\mu_A(t)]}{n-t}. \end{aligned}$$

Now consider the second term $E\left[(A(\mathcal{T}) - A(\mathcal{S}))^2\right]$. This is the expected squared bias due to using a training set of size $t < n$ to estimate the accuracy of a classifier developed on the full dataset of size n. This squared bias could be estimated using Monte Carlo in restricted simulation settings where the exact accuracies $A(\mathcal{T})$ and $A(\mathcal{S})$ can be calculated mathematically for every

Monte Carlo simulation. However, in more realistic settings, $A(\mathcal{T})$ and $A(\mathcal{S})$ cannot be calculated because the distribution of the populations in unknown. In order to develop a more general approach, it is useful to instead further decompose this expected mean squared error as follows:

$$\begin{aligned} E\left[(A(\mathcal{T}) - A(\mathcal{S}))^2\right] &= E\left[(A(\mathcal{T}) - \mu_A(t) + \mu_A(t) - \mu_A(n) + \mu_A(n) - A(\mathcal{S}))^2\right] \\ &\propto E\left[(A(\mathcal{T}) - \mu_A(t))^2\right] + (\mu_A(t) - \mu_A(n))^2 \\ &= A + B \end{aligned}$$

where, in the final line, we have denoted A as the accuracy variance for samples of size t, and B as the squared bias term. Note that the proportionality in going from line 1 to line 2 comes from the fact that the third term in the equation does not involve t.

Now these decompositions above involve assumptions that the cross-terms are negligible. This assumption can be partially evaluated in the simulation setting by comparing the MSE patterns as the training size varies, where the comparison is between the MSE pattern estimated using the decomposition above to the MSE pattern from brute force Monte Carlo.

1.3 A justification for the rule-of-thumb suggestion

The motivation for this recommendation is easiest to see by considering situations in which this is a *bad idea*, because $n/2$ to training results in much lower mean squared error (MSE) than $2n/3$. Then, by showing that these situations are unlikely to happen when the full dataset accuracy is over 85%, the conclusion naturally follows that in situations that are likely to happen, $2n/3$ to the training set is a safe and robust strategy.

The mean squared error can be broken down into $MSE = V + A + B$. We consider these terms in the order B, V, and A.

B, the squared bias : The squared bias term will never be larger at $t = 2n/3$ than it is at $t = n/2$, except in the unlikely case that the mean accuracy *decreases* as the training set sample size increases. This could only happen with a poorly chosen predictor development algorithm.

V, the binomial variance In all our simulations, this was the key term that dominated when t approached n. However, the difference between the V term at $n/2$ and at $2n/3$ term is bounded above by $\frac{1}{4n}$. For example, if $n = 100$, then the difference is ≤ 0.0025. In order for this term to be much larger at $n/2$ than $2n/3$, n needs to be fairly small[1].

[1]That being said, in the simulations, when n was small and the performance on the full dataset was $> 60\%$, the best performing predictor tended to have *more* samples assigned to training.

A, the accuracy variance The accuracy variance stayed relatively flat in all our simulations. Given the preceding considerations, the most feasible scenario in which $n/2$ would result in a much smaller MSE than $2n/3$ would be a setting in which the accuracy variance at $2n/3$ was much larger than at $n/2$. Importantly, accuracy variance that increases significantly with sample size would be an undesirable property in a predictor development algorithm. While not inconceivable, this would contradict what was observed in the simulation datasets and the real datasets examined. But there could be predictors that behave this way. This possibility could be explored through simulation or parametric bootstrap.

In conclusion, our rule-of-thumb advice is to use 2/3rds of the samples for the training set when the full dataset accuracy is believed to be over 85%. The key assumptions for this general rule-of-thumb are that 1) the accuracy variance at $t = 2n/3$ will not be much larger than at $t = n/2$. The key assumption (1) should generally be true, but can also be evaluated to some extent by simulations using the predictor development algorithm that is to be used in the proposed study.

2 Supplemental Section 2: Table S1

		**n = 200**			
**m = 50**	$t:v$<br>$\bar{A}(n)$	170:30<br>(83%)	70+:130-<br>(> 99%)	40+:160-<br>(> 99%)	20+:180-<br>(> 99%)
**m = 10**	$t:v$<br>$\bar{A}(n)$	150:50<br>(62%)	150:50<br>(94%)	90:110<br>(99%)	40+:160-<br>(> 99%)
**m = 1**	$t:v$<br>$\bar{A}(n)$	10:190<br>(51%)	150:50<br>(68%)	130:70<br>(77%)	90:110<br>(84%)
		$\mathbf{2\delta/\sigma = 0.5}$	$\mathbf{2\delta/\sigma = 1.0}$	$\mathbf{2\delta/\sigma = 1.5}$	$\mathbf{2\delta/\sigma = 2.0}$
		**n = 100**			
**m = 50**	$t:v$<br>$\bar{A}(n)$	70:30<br>(65%)	80+:20-<br>(> 99%)	40+:60-<br>(> 99%)	20+:80-<br>(> 99%)
**m = 10**	$t:v$<br>$\bar{A}(n)$	10:90<br>(53%)	80:20<br>(89%)	70:30<br>(99%)	40+:60-<br>(> 99%)
**m = 1**	$t:v$<br>$\bar{A}(n)$	10:90<br>(50%)	40:60<br>(60%)	80:20<br>(76%)	70:30<br>(84%)
		$\mathbf{2\delta/\sigma = 0.5}$	$\mathbf{2\delta/\sigma = 1.0}$	$\mathbf{2\delta/\sigma = 1.5}$	$\mathbf{2\delta/\sigma = 2.0}$
		**n = 50**			
**m = 50**	$t:v$<br>$\bar{A}(n)$	10:40<br>(56%)	40:10<br>(96%)	40:10<br>(> 99%)	20+:30-<br>(> 99%)
**m = 10**	$t:v$<br>$\bar{A}(n)$	10:40<br>(51%)	40:10<br>(70%)	40:10<br>(97%)	40:10<br>(> 99%)
**m = 1**	$t:v$<br>$\bar{A}(n)$	10:40<br>(50%)	10:40<br>(53%)	20:30<br>(64%)	40:10<br>(80%)
		$\mathbf{2\delta/\sigma = 0.5}$	$\mathbf{2\delta/\sigma = 1.0}$	$\mathbf{2\delta/\sigma = 1.5}$	$\mathbf{2\delta/\sigma = 2.0}$

Table S1: Entries show the split of the sample that minimizes the mean squared error (MSE). Unequal prevalence from each of two classes, namely, 67% from one class and 33% from the other class. Total sample size is n. Table entries $t : v$ indicate the optimal allocation to the training t and validation v sets, with $n = t + v$. The average accuracy estimate for the sample size of n is $\bar{A}(n)$. m is the number of differentially expressed genes, each differentially expressed by a standardized fold change of $2\delta/\sigma$. Table notation such as "50+:150-" indicates that the MSE was flat, achieving a minimum at t=50 and remaining at that minimum for $t > 50$. Here, "flat" is defined as having a range of MSE values with $|MSE_{max} - MSE_{min}| < 0.0001$. $P = 22,000$ genes on arrays. Each table entry based on $1,000$ Monte Carlo simulations.

3 Supplemental Section 3: Figures from the model-based approach

3.1 Equal prevalence (50% from each class) simulations

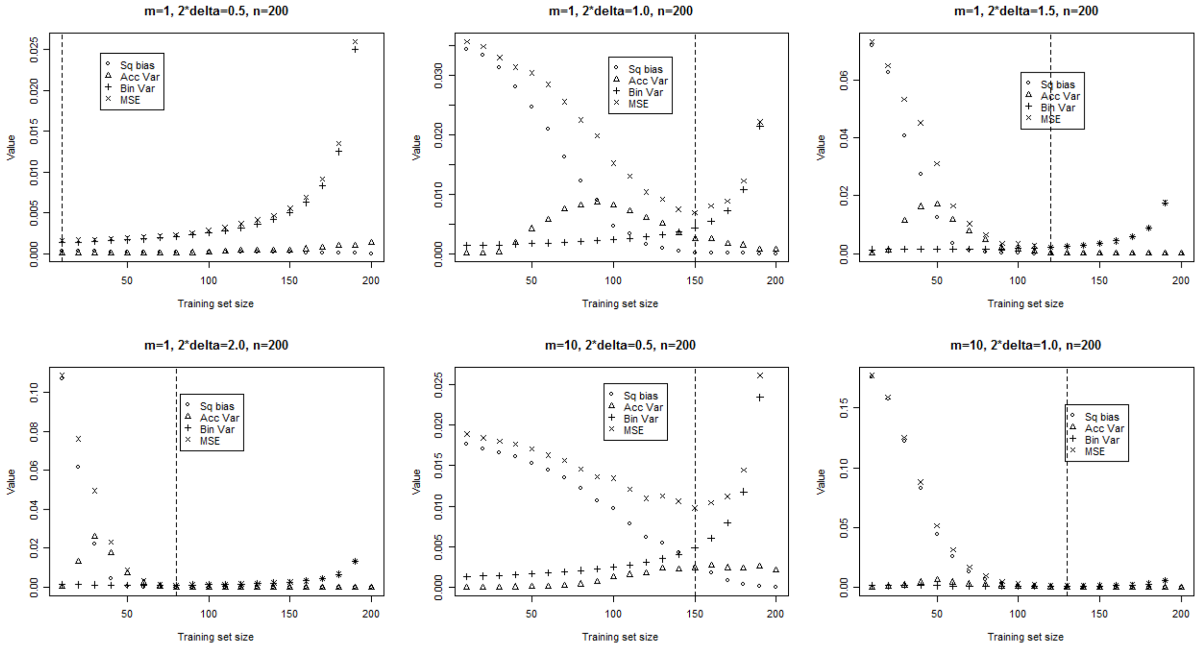

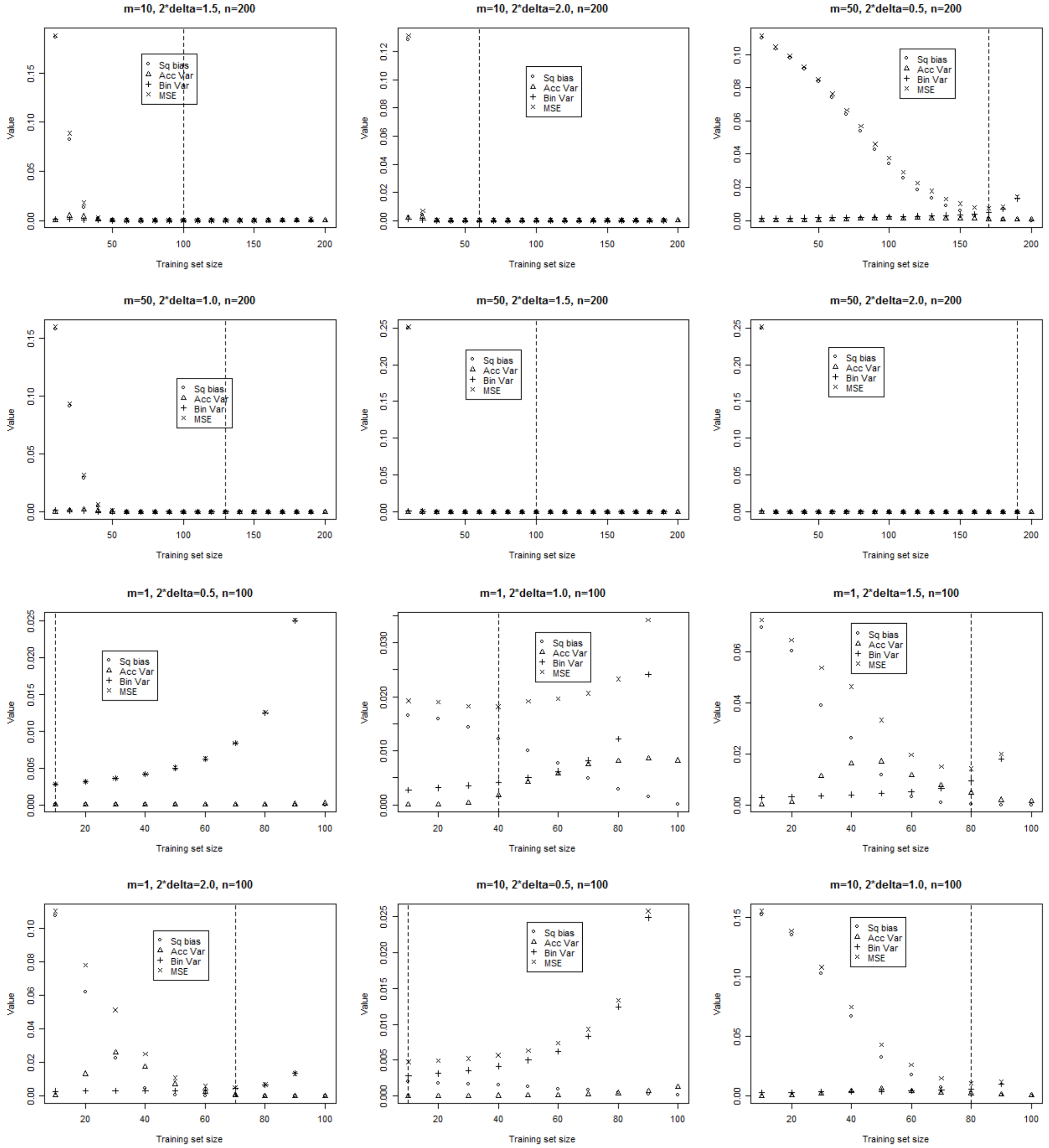
m=10, 2*delta=1.5, n=200
m=10, 2*delta=2.0, n=200
m=50, 2*delta=0.5, n=200
m=50, 2*delta=1.0, n=200
m=50, 2*delta=1.5, n=200
m=50, 2*delta=2.0, n=200
m=1, 2*delta=0.5, n=100
m=1, 2*delta=1.0, n=100
m=1, 2*delta=1.5, n=100
m=1, 2*delta=2.0, n=100
m=10, 2*delta=0.5, n=100
m=10, 2*delta=1.0, n=100
Sq bias
Acc Var
Bin Var
MSE
Value
Training set size

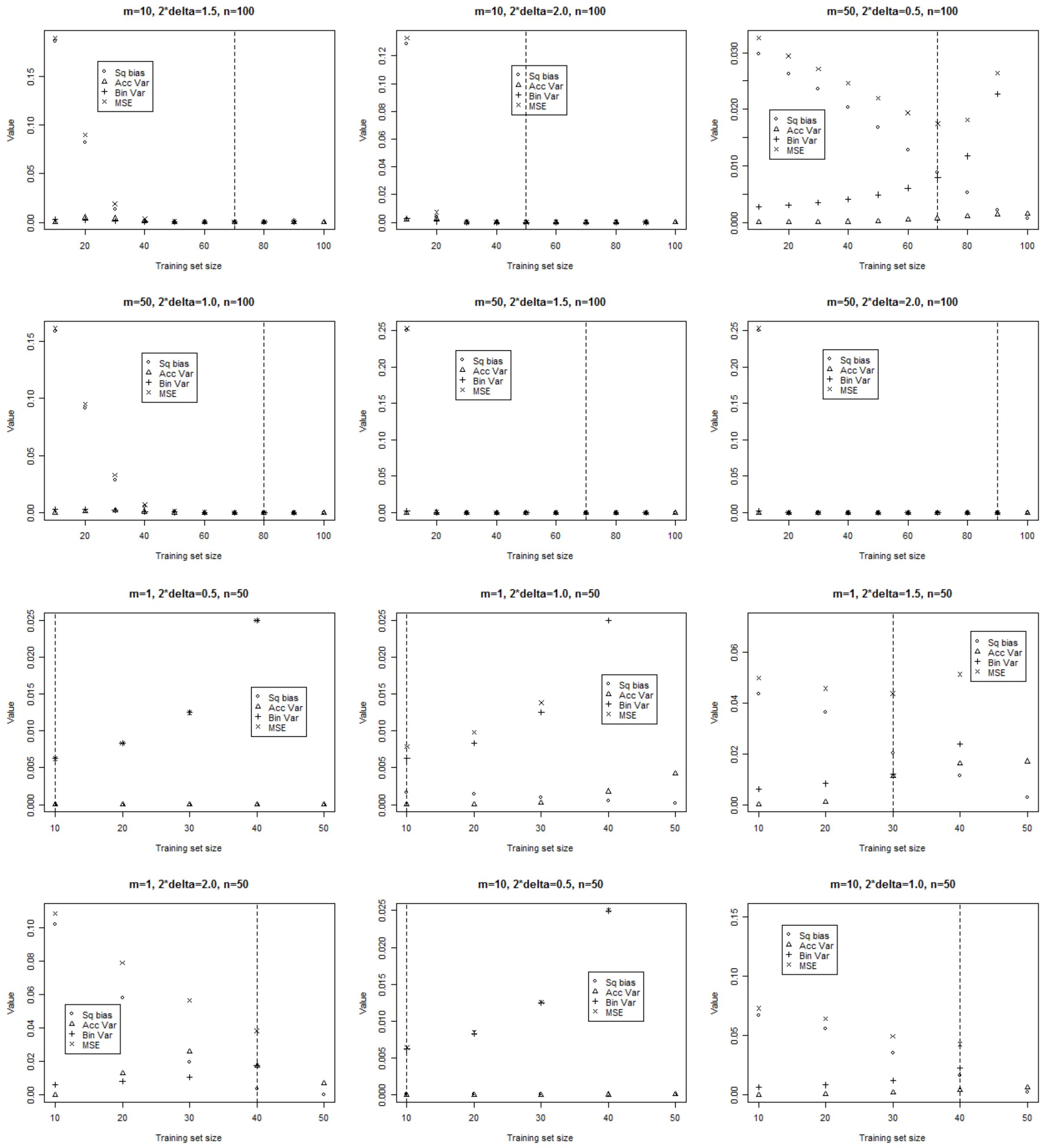
m=10, 2*delta=1.5, n=100
m=10, 2*delta=2.0, n=100
m=50, 2*delta=0.5, n=100
m=50, 2*delta=1.0, n=100
m=50, 2*delta=1.5, n=100
m=50, 2*delta=2.0, n=100
m=1, 2*delta=0.5, n=50
m=1, 2*delta=1.0, n=50
m=1, 2*delta=1.5, n=50
m=1, 2*delta=2.0, n=50
m=10, 2*delta=0.5, n=50
m=10, 2*delta=1.0, n=50
Sq bias
Acc Var
Bin Var
MSE
Value
Training set size

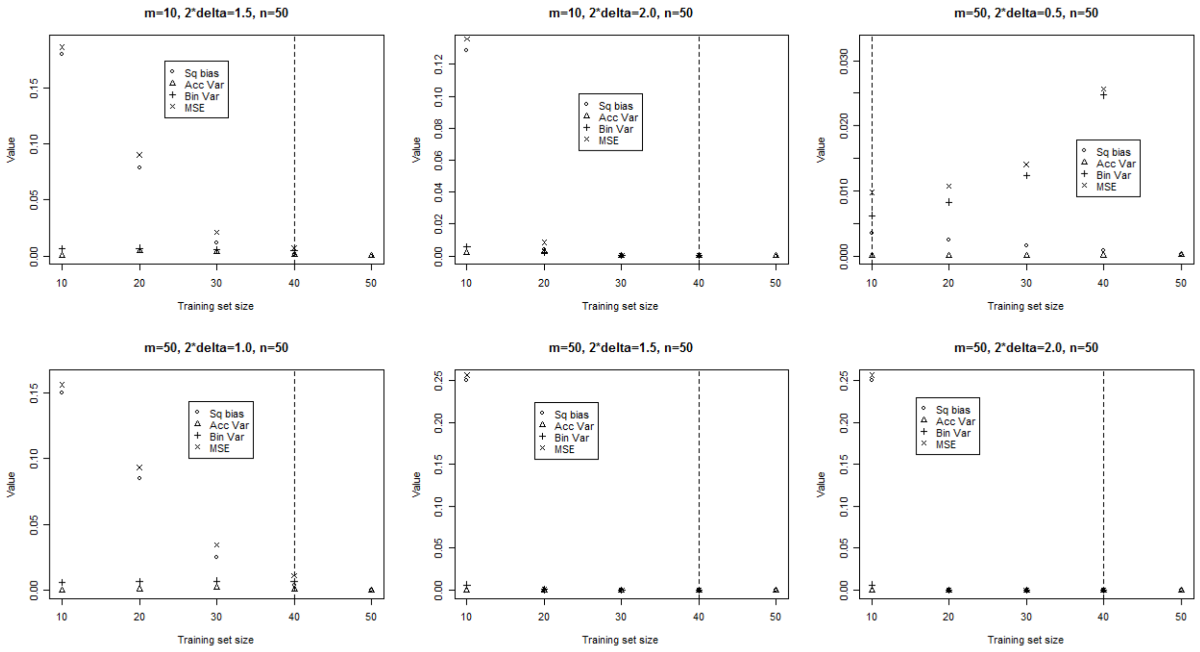
m=10, 2*delta=1.5, n=50
m=10, 2*delta=2.0, n=50
m=50, 2*delta=0.5, n=50
m=50, 2*delta=1.0, n=50
m=50, 2*delta=1.5, n=50
m=50, 2*delta=2.0, n=50
Sq bias
Acc Var
Bin Var
MSE
Value
Training set size

3.2 Unequal class prevalence (67% versus 33%) simulations

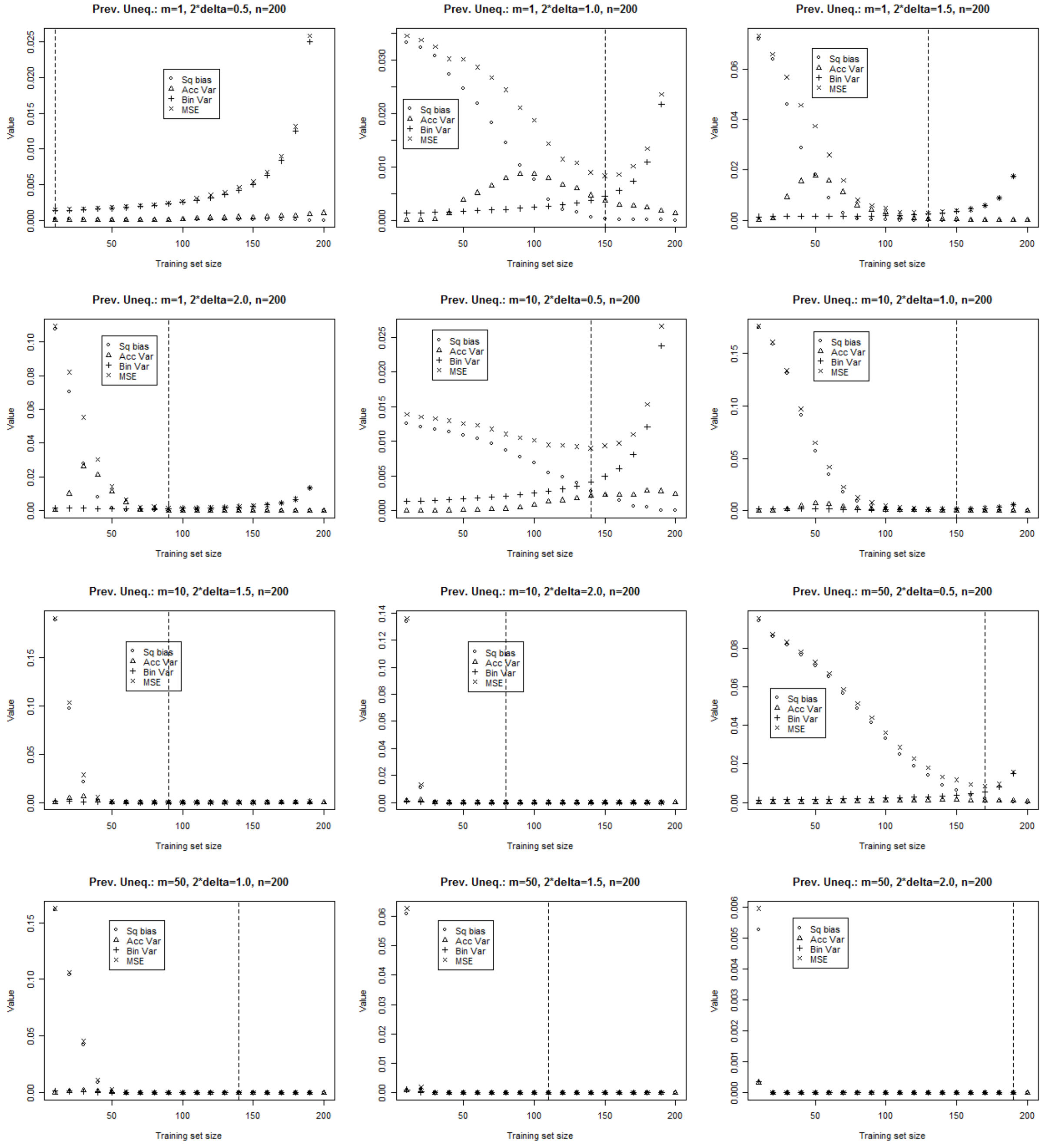

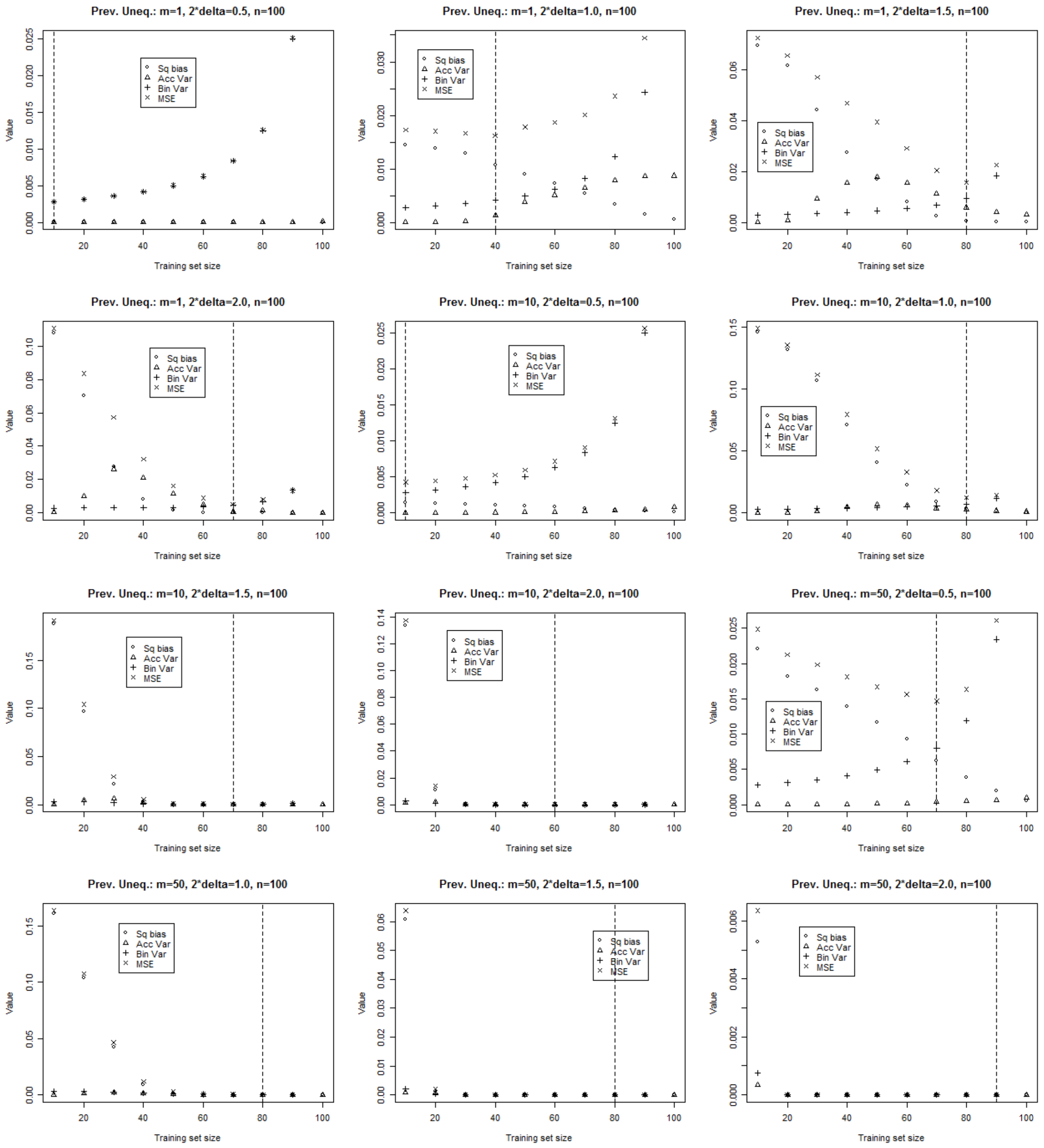
Prev. Uneq.: m=1, 2*delta=0.5, n=100
Prev. Uneq.: m=1, 2*delta=1.0, n=100
Prev. Uneq.: m=1, 2*delta=1.5, n=100
Prev. Uneq.: m=1, 2*delta=2.0, n=100
Prev. Uneq.: m=10, 2*delta=0.5, n=100
Prev. Uneq.: m=10, 2*delta=1.0, n=100
Prev. Uneq.: m=10, 2*delta=1.5, n=100
Prev. Uneq.: m=10, 2*delta=2.0, n=100
Prev. Uneq.: m=50, 2*delta=0.5, n=100
Prev. Uneq.: m=50, 2*delta=1.0, n=100
Prev. Uneq.: m=50, 2*delta=1.5, n=100
Prev. Uneq.: m=50, 2*delta=2.0, n=100
Sq bias
Acc Var
Bin Var
MSE
Value
Training set size

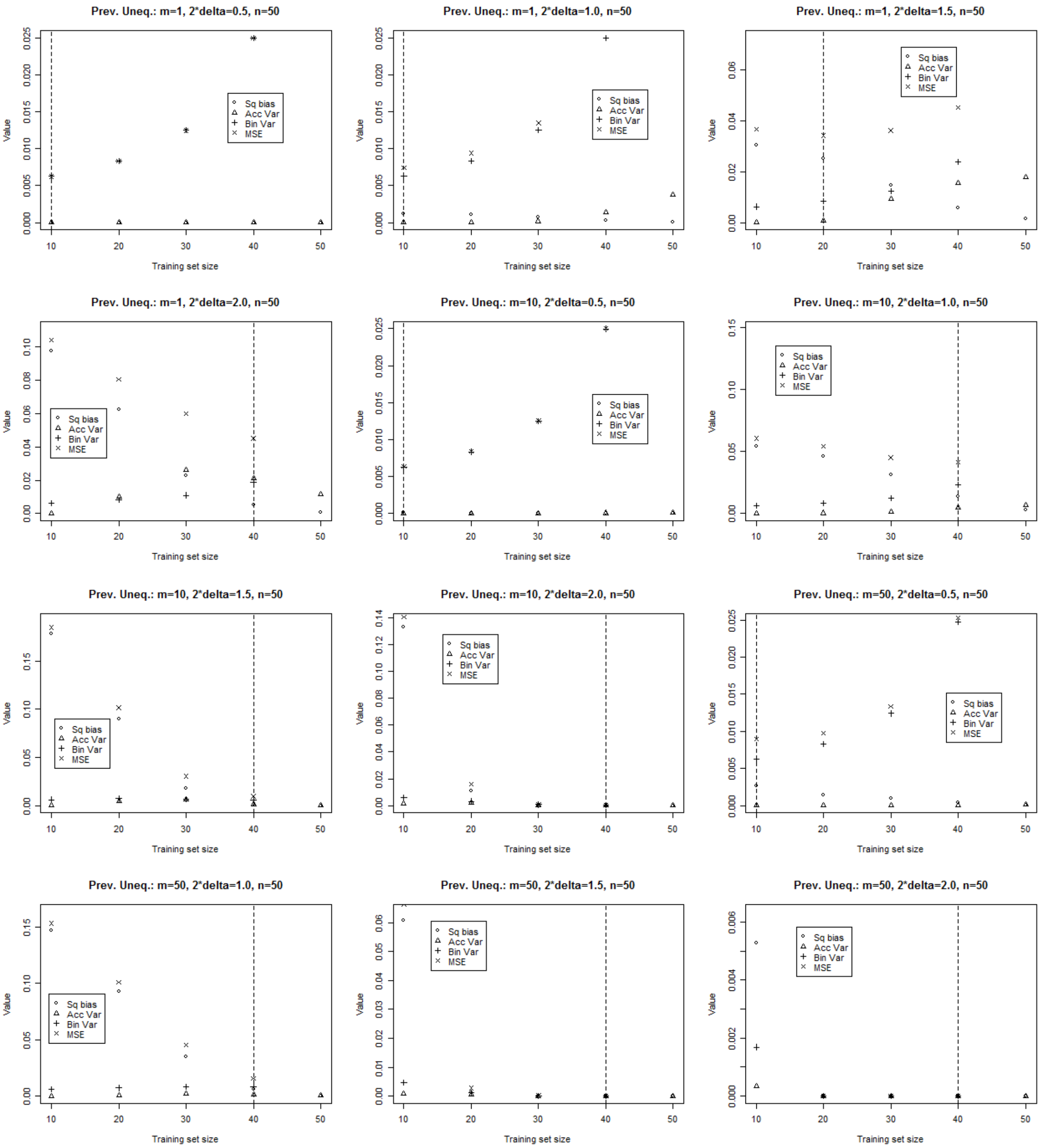
Prev. Uneq.: m=1, 2*delta=0.5, n=50
Prev. Uneq.: m=1, 2*delta=1.0, n=50
Prev. Uneq.: m=1, 2*delta=1.5, n=50
Prev. Uneq.: m=1, 2*delta=2.0, n=50
Prev. Uneq.: m=10, 2*delta=0.5, n=50
Prev. Uneq.: m=10, 2*delta=1.0, n=50
Prev. Uneq.: m=10, 2*delta=1.5, n=50
Prev. Uneq.: m=10, 2*delta=2.0, n=50
Prev. Uneq.: m=50, 2*delta=0.5, n=50
Prev. Uneq.: m=50, 2*delta=1.0, n=50
Prev. Uneq.: m=50, 2*delta=1.5, n=50
Prev. Uneq.: m=50, 2*delta=2.0, n=50
Value
Training set size
Sq bias
Acc Var
Bin Var
MSE

4 Supplemental Section 4: Comparison of model-based and bootstrap approach on the same simulated datasets.

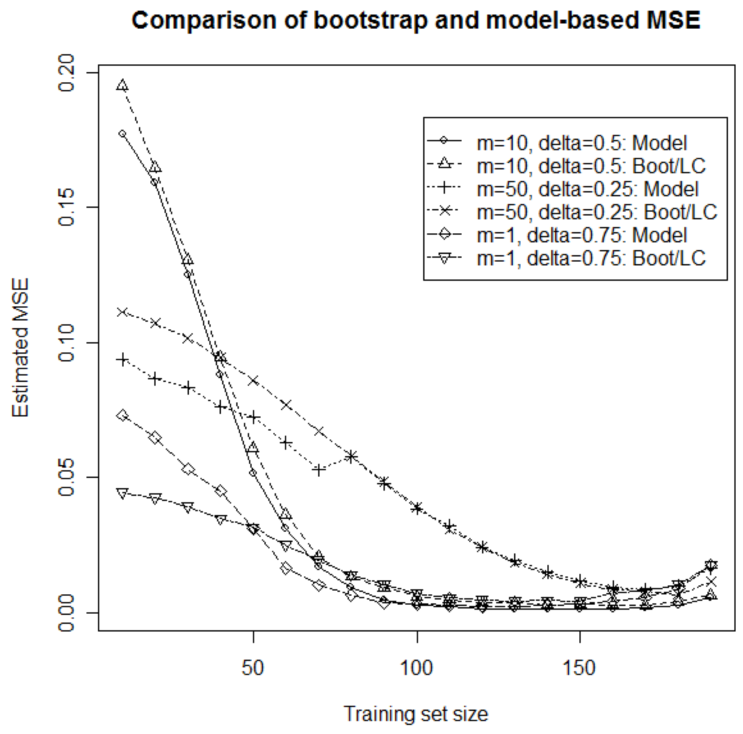

5 Supplemental Section 5: Empirically estimated covariance and effects simulations

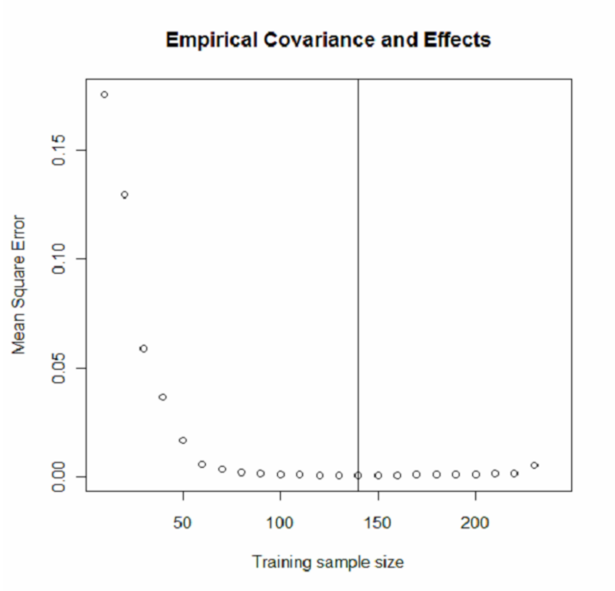

Figure to correspond to empirical covariance and effects simulation with $p = 0.9$ *and* $PCC(n = 240) = 96\%$.

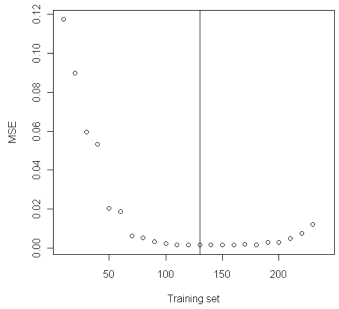

Figure to correspond to empirical effects and covariance simulation with $p = 0.6$ *and* $PCC(n = 240) = 86\%$.

6 Supplemental Section 6: Analysis of microarray datasets

6.1 Golub et al. dataset

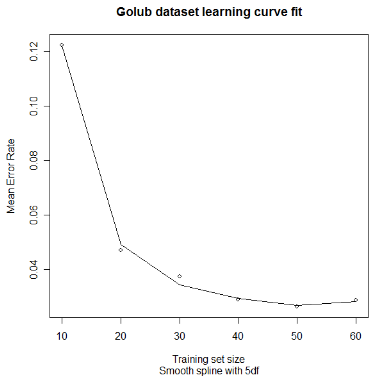

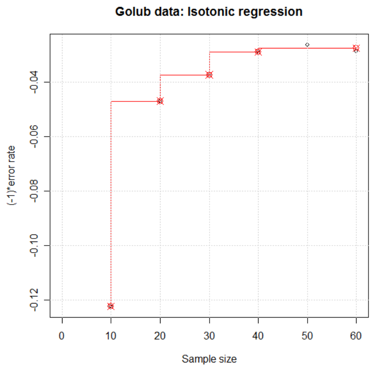

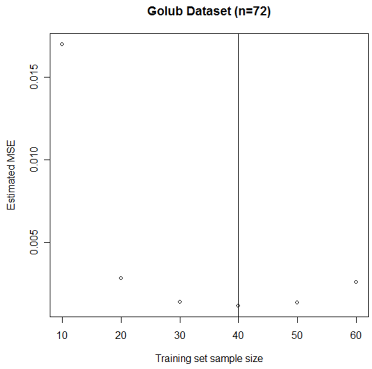

Golub estimated MSE based on 1000 bootstraps to estimate the variance term, and 1000 sample splits to estimate the squared bias term. Isotonic regression results used for the MSE figure above.

6.2 Rosenwald et al. data

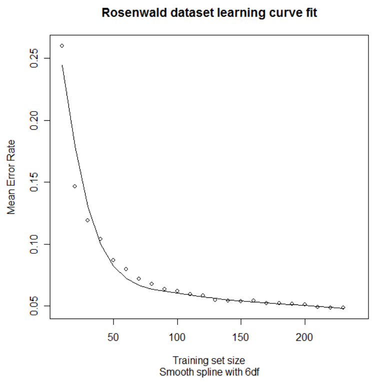

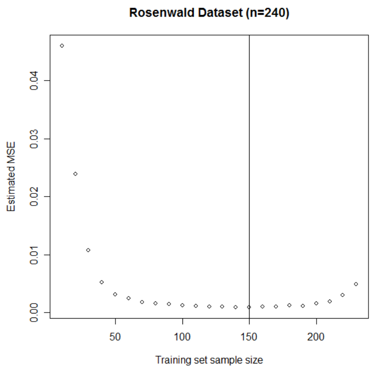

6.3 Boer et al. data

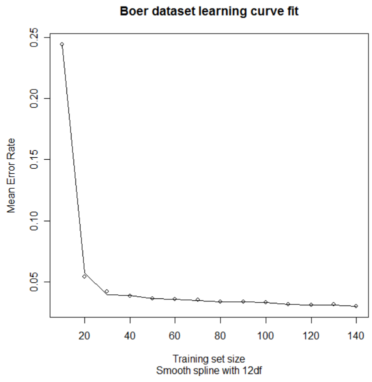

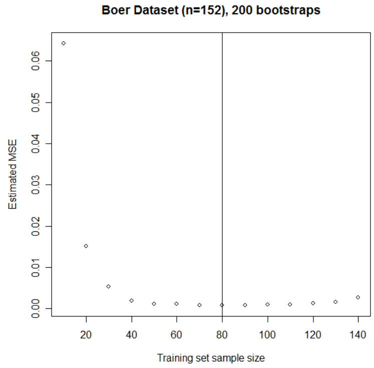

6.4 Sun et al. data

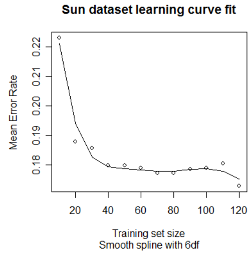

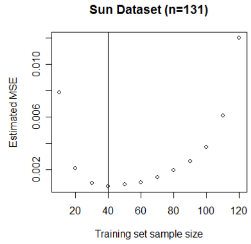

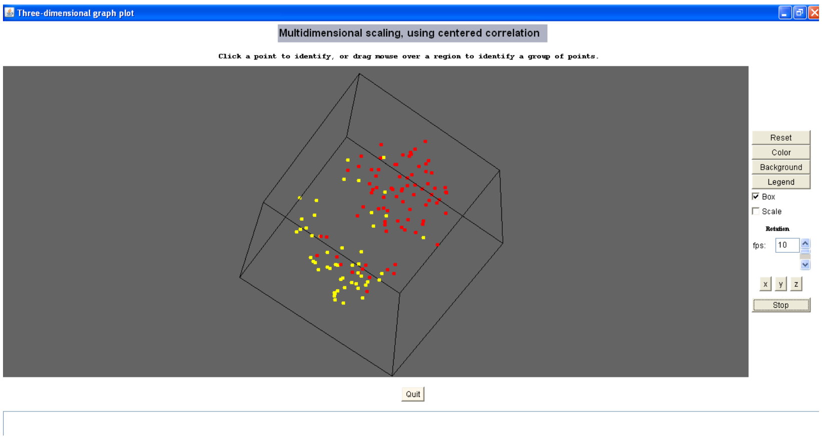

Above: Sun dataset multidimentional scaling plot, colored by sample type (oligodendroglioma versus glioblastoma).

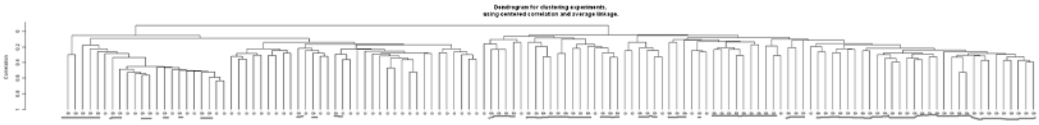

Above: Sun dataset clustering dendrogram, marked by class, showing 2 strong clusters.

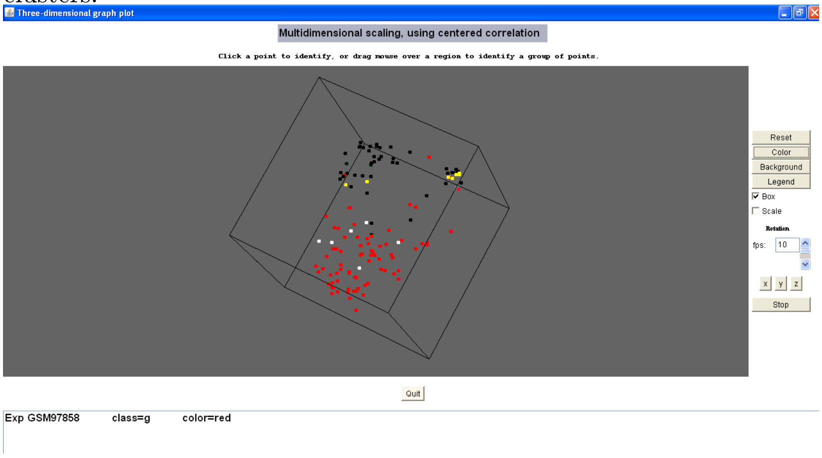

Above: Sun dataset. Red: Glioblastoma Black: Oligodendroglioma Yellow: Glioblastomas incorrectly called by three predictors developed on samples of size n=20. White: Oligodendrogliomas incorrectly called by three predictors developed on samples of size n=20.

6.5 van't Veer et al

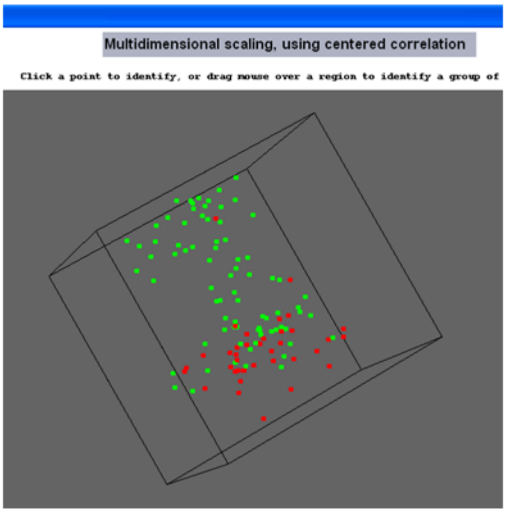

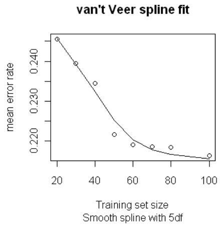

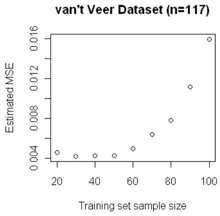

Supplement: Additional file 1 — Article supplement. Contains additional tables, figures, theoretical derivations and discussions. [file 1755-8794-4-31-S1.PDF]
